# Circulating Tumor DNA Testing Supports Rapid Therapeutic Decision-Making in Metastatic Melanoma: A Case Report

**DOI:** 10.3389/fonc.2022.846187

**Published:** 2022-06-10

**Authors:** Tapas Ranjan Behera, Jung Min Song, Jennifer Ko, Donald Eicher, Joshua Arbesman, Brian Gastman, Daniel H. Farkas, Pauline Funchain

**Affiliations:** ^1^Taussig Cancer Institute, Cleveland Clinic, Cleveland, OH, United States; ^2^Anatomic Pathology, Cleveland Clinic, Cleveland, OH, United States; ^3^ Dermatology & Plastic Surgery Institute, Cleveland Clinic, Cleveland, OH, United States; ^4^Pathology and Laboratory Medicine Institute, Cleveland Clinic, Cleveland, OH, United States

**Keywords:** Rapid ctDNA test, ctDNA, *BRAF* mutation, rt-PCR, targeted therapy, melanoma

## Abstract

Treatment of metastatic melanoma includes the option of targeted therapy in patients with driver *BRAF* mutations. BRAF-MEK inhibitor drugs improve survival in the approximately 50% of patients with melanoma that harbor *BRAF* mutations. As *BRAF* mutation detection in tissue often takes days to weeks, it is not always possible or timely to obtain *BRAF* status in tissue using immunohistochemistry or next generation sequencing. Plasma-derived circulating tumor DNA (ctDNA) is a potential alternative analyte in such treatment settings. We present a case of metastatic melanoma that was treated in an emergent setting using therapy supported by rapid PCR-based detection of ctDNA positive for a *BRAF* V600 mutation. In this rapidly deteriorating 53-year-old male with diffuse melanoma metastases and unknown *BRAF* mutation status requiring hospital admission, a plasma-based *BRAF* mutation detection supported treatment with targeted therapy, dabrafenib and trametinib. Same-day initiation of therapy resulted in swift amelioration allowing discharge within a week, followed by substantial clinical improvement over the following weeks. In cases requiring urgent clinical decision making, a plasma-based, near point-of-care detection system is useful in supporting targeted therapy decisions without the need for invasive and time-consuming biopsy.

## Introduction

Circulating tumor DNA (ctDNA) is a useful disease monitoring analyte in many cancers (1). Diagnosing cancer from evaluation of ctDNA has been reported in lung and trophoblastic tumors (2–4). Generally, definitive diagnosis employs early imaging-based identification and/or specific laboratory testing, or pathology investigation. ctDNA evaluation is not usually employed diagnostically. Furthermore, since ctDNA tends to appear in detectable levels in the peripheral blood in advanced disease, the utility of ctDNA for impactful characterization in advanced cancer is clinically attractive, especially when detection of specific markers can affect therapy choice and thus clinical outcome.

Melanoma is a rapidly progressive cancer that, when detected at advanced stages, limits the efficacy of available treatment options. The identification of *BRAF* mutation status in melanoma offers the option for specific targeted therapy agents. Specifically, the combination of BRAF and MEK inhibitors in melanoma are highly effective in improving survival and often precipitate rapid symptom resolution (5). Typically, surgical resection of melanoma for histopathological diagnosis generates a specimen for evaluation of *BRAF* mutation status by tissue immunohistochemistry (IHC) or molecular diagnostic techniques, which is accomplished by next generation sequencing (NGS). Tissue-based *BRAF* detection may take days to weeks, depending on an institution’s capabilities and whether tests are typically “sent out” to a reference laboratory. In patients where the primary site is occult, and sites of metastases are not accessible for tissue sampling, *BRAF* mutation status may be difficult to obtain and/or remain unknown. In advanced melanoma, ctDNA is generally higher in concentration compared to earlier stages (6). For patients needing urgent *BRAF* status evaluation to aid clinical decision-making regarding therapy choice(s), a PCR-based, near-point of care, rapid testing platform could be useful to detect *BRAF* variants in plasma-derived ctDNA (7).

## Case Presentation

A 53-year-old male presented to an outside hospital with a three-week history of nausea, vomiting, and jaundice. On admission, he complained of abdominal discomfort, back pain, jaundice, and dark urine. He was afebrile, normotensive, with non-cholangitic pain. The patient was admitted for severe jaundice in face of a suspected metastatic process. On admission, creatinine was 1.38, and LFTs were elevated; total bilirubin was 21.9, ALP 359, ALT 211, AST 202, lipase normal, lactate 2.7 (**Table 1**). Chest X-ray redemonstrated a previously identified chest nodule of 3.5 x 2.5 cm in the mid to lower left lung without any consolidation or effusion. An abdominal radiograph did not show dilated bowel loops. CT scan of the abdomen identified widespread metastatic disease in the liver, a 2.5 cm renal mass in left lower pole, and a non-specific sclerotic focus in the right ileum (**Figure 2**). CT scan of the chest identified a lingular mass (**Figure 3**), bilateral pulmonary nodules, and a destructive lesion in the T3 vertebral body. Pathologic review of a liver biopsy from an outside hospital revealed metastatic melanoma. An MRI of the brain was negative for intracranial or leptomeningeal metastases. Due to the need for rapid assessment of BRAF status for therapeutic decision making, the patient underwent another liver biopsy for evaluation of *BRAF* mutation status. Simultaneously, a plasma sample was tested for ctDNA *BRAF* mutation status.

**Table 1:** Patient laboratory values pre-treatment at admission (day 0) and post-treatment on day 6, 19, and 28.

Laboratory Parameter	DAY 0 admission	Day 6 discharge	Day 19 visit	Day 28 visit
**BUN (9-24mg/dl)**	50	47	11	13
**Creatinine (0.73-1.22 mg/dl)**	1.38	1.20	0.87	0.92
**Bilirubin, Total (0.2-1.3 mg/dl)**	21.9	21.3	7.8	5.1
**Alkaline Phos. (38-113 U/l)**	359	315	324	278
**ALT (10-54 U/l)**	211	158	64	36
**AST (14-40 U/l)**	202	142	69	46
**Anion Gap (9-18 mmol/l)**	15	11	5	9
**LD (135-225 U/l)**		2667 (on day 4)	1179	840 (on day 32)

Treatment with BRAF MEK inhibitors was initiated on day 5 from hospital admission.

**Figure 1:**
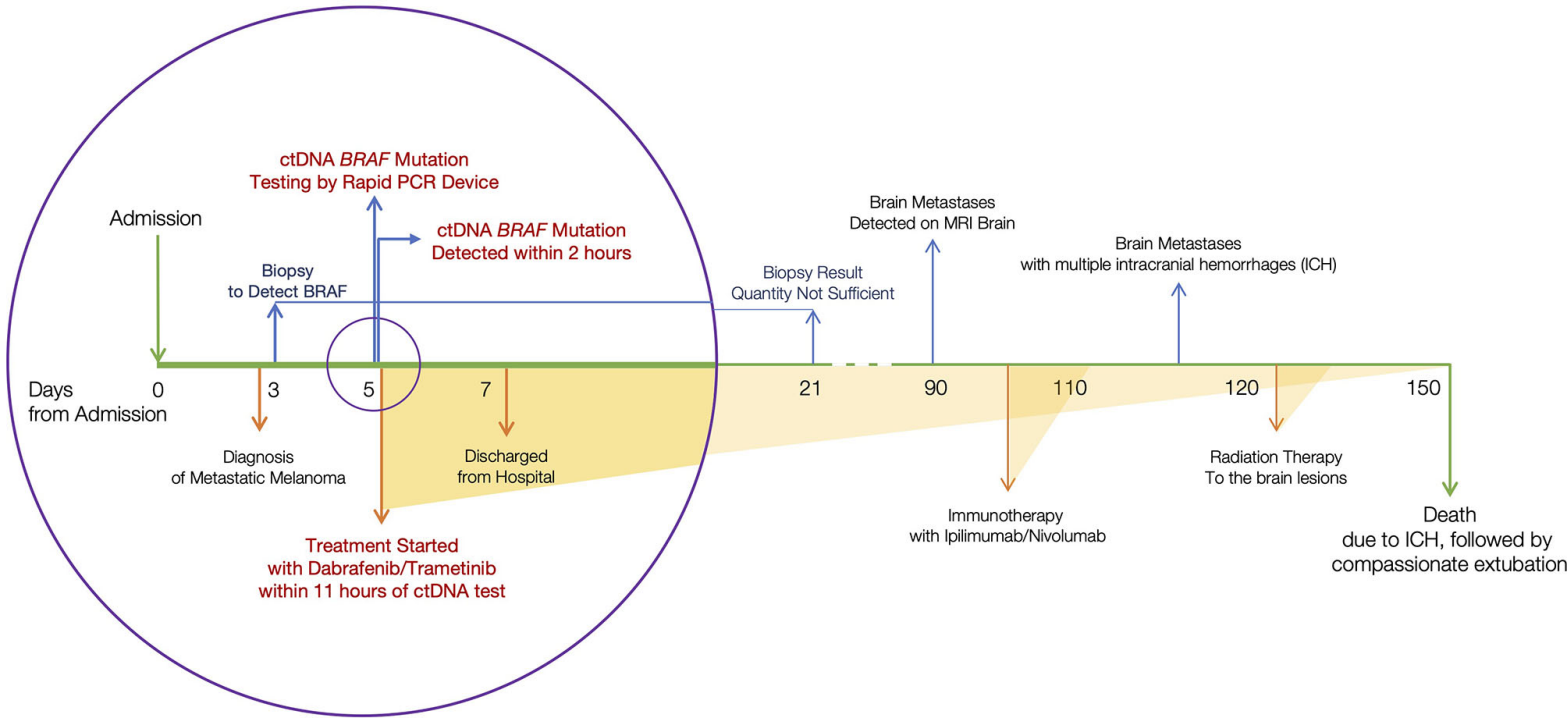
Timeline of clinical events. Considering the day of admission as day 0, ctDNA *BRAF* variant was identified on day 5. Therapy was initiated the same day.

The plasma sample tested positive for ctDNA-based *BRAF*. Supported by this ctDNA finding, BRAF/MEK inhibitors, dabrafenib and trametinib, were initiated. Patient lab values improved within two weeks of treatment initiation; bilirubin levels decreased from 21.3 to 7.8 mg/dL. The patient’s clinical condition improved and he was discharged from the hospital within a week of admission. The patient’s treatment events during admission are represented in **Figure 1** (circle).

**Figure 2:**
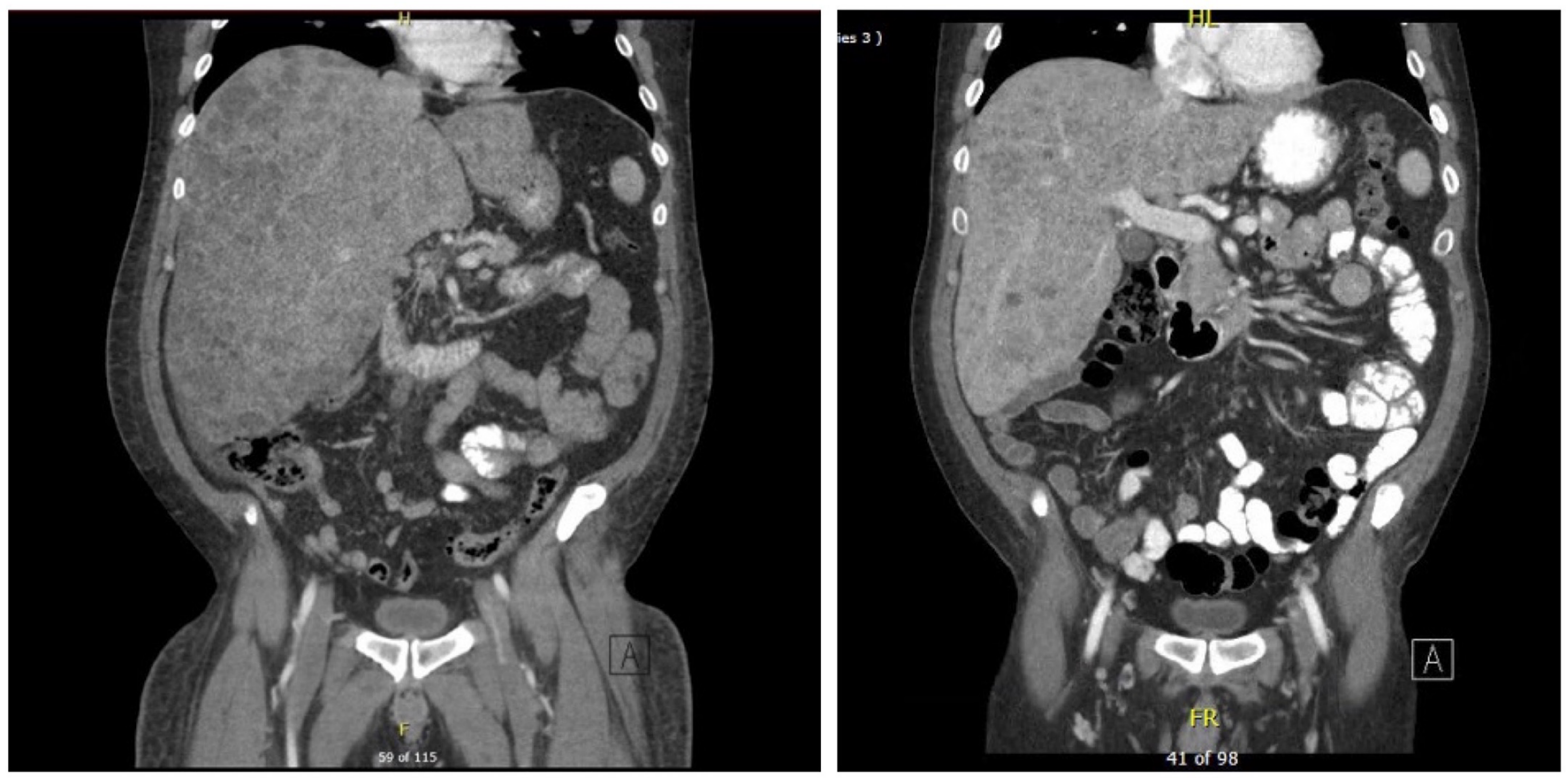
CT scan of abdomen showing innumerable lesions throughout the liver consistent with metastatic disease on day 1 on the left vs day 104 on the right with improved lesion sizes.

**Figure 3:**
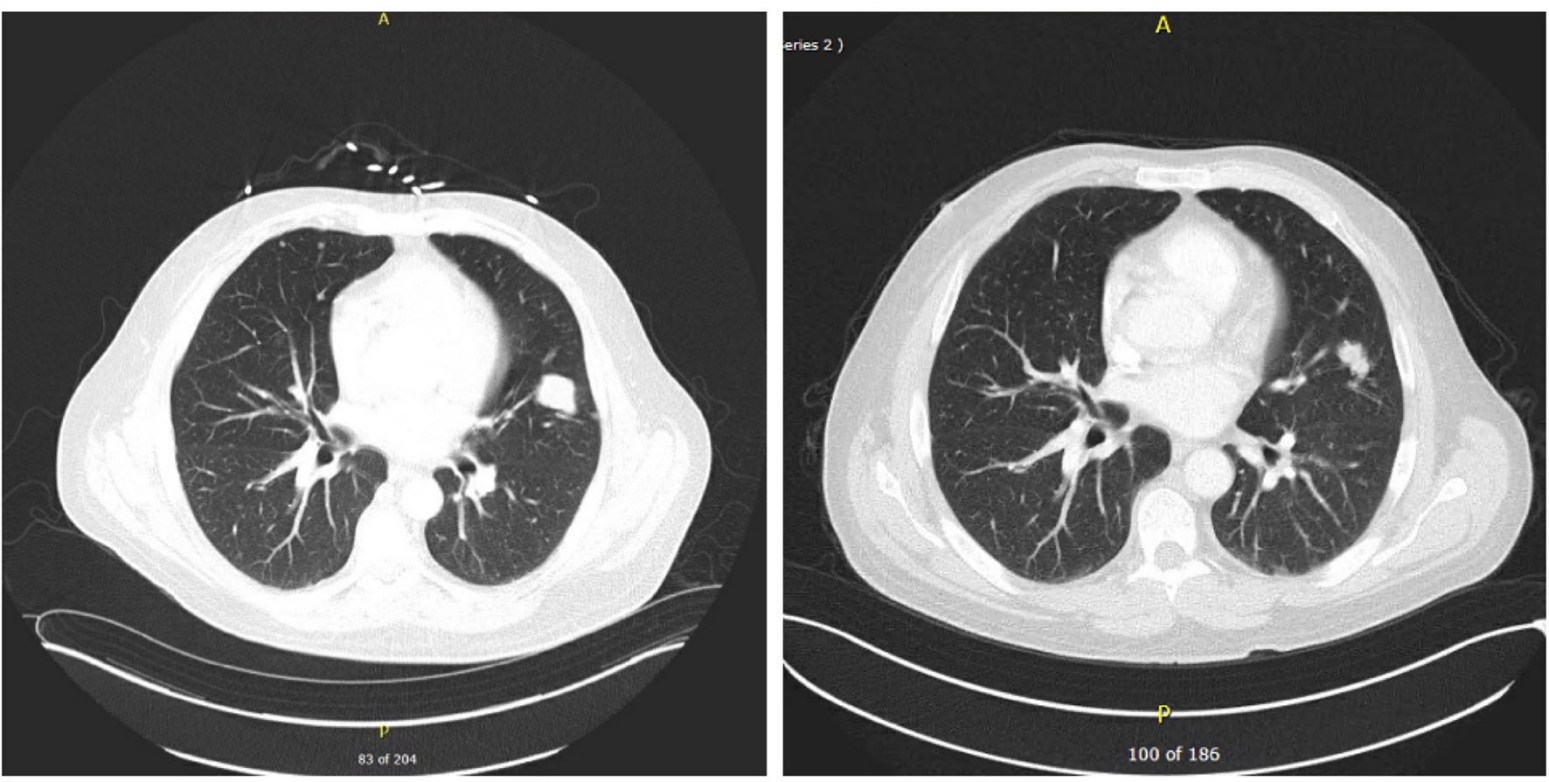
CT scan of chest showing dominant lingular mass concerning for primary lung cancer or metastatic lesion. Multiple bilateral small pulmonary nodules and T3 vertebral body destructive lesion seen concerning for metastatic disease on day 1 on the left vs on day 104 on the right showing decrease in sizes and numbers of pulmonary nodules suggestive of favorable treatment response.

CT scans of the abdomen/pelvis and chest showed significant response to the therapy as shown in **Figures 2** and **3**. The imaging on day 104, compared to that of day one, demonstrated decreased size and number of innumerable hepatic masses. For example, a 1.6 x 1.3 cm right dome lesion, previously measured 5.1 x 4.0 cm. Lymph node sizes also decreased; left periaortic node from 1.7 x 1.2 to 1.4 x 0.8 cm; aortocaval node from 1.4 x 1.3 to 1.2 x 1.0 cm; and caval node from 1.8 x 1.5 to 1.9 x 1.3 cm. The sizes of the peritoneal implants also decreased from 1.5 x 1.1 cm to 1.2 x 0.7 cm. Chest imaging showed significant decreases in size and number of multiple scattered bilateral pulmonary nodules, suggestive of metastatic disease with favorable treatment response.

Serial ctDNA-based variant levels were assessed over time and remained consistently detectable. Three months after the initial admission, an MRI scan of the brain demonstrated multiple small lesions of the bilateral frontal lobes and left occipital lobe. Radiation therapy was initiated but the patient’s condition deteriorated when multiple hemorrhagic metastases developed seven weeks after initial metastasis detection. The patient was initially intubated, and compassionately extubated when no medical options for improvement remained.

## Discussion

This is, to our knowledge, the first reported case where the clinical decision for treatment in melanoma was supported by the result of rapid ctDNA-based *BRAF* variant identification. The case demonstrates the impact rapid ctDNA-based variant detection can make when a tissue biopsy is not available or when awaiting biopsy results can lead to treatment delay, particularly in a quickly declining, admitted patient. Combination BRAF/MEK inhibitors can result in very rapid amelioration of a patient’s clinical condition. Immediate onset of positive drug effects can be seen in some clinical situations, thus, having access to a rapid test for *BRAF* variants in melanoma can dramatically affect time to treatment initiation, an important variable in progression-free and overall survival (8).

Tumor biopsy is the conventional source of tissue for *BRAF* variant interrogation in melanoma. Next Generation Sequencing (NGS) performed on the biopsy tissue is the gold standard for detecting BRAF mutation in patients diagnosed with melanoma. Tissue immunohistochemistry (IHC) is a sensitive and relatively quicker alternative routinely employed to identify the presence of BRAF V600E mutations (9). While NGS and IHC rely on availability of biopsy tissue, a liquid biopsy, *i.e.*, examination of ctDNA in plasma specimens, may serve as an alternative when tissue specimens cannot be obtained, or when insufficient tumor tissue is available. Furthermore, liquid biopsy could provide a result in as early as an hour compared to days to weeks in tests employing NGS or IHC. In this case, we showed that liquid biopsy was useful when treatment decisions must be made quickly. It can take days to get relevant NGS or immunohistochemistry results, depending on laboratory case load, “send-out” logistics, etc. In this case, a real-time PCR-based, automated testing device (Idylla; Biocartis, Belgium) was employed to detect *BRAF* variants in plasma-derived ctDNA (10). The test requires very little sample input and minimal technical demand; one mL of plasma is introduced into a testing cartridge which is inserted into the instrument. The assay is semi-quantitative in nature; positive results are based on a minimum detectable variant threshold level. Test results are binary – positive or negative; V600E, V600E2, and V600D are simultaneously interrogated and not differentiated in the analysis. From the time blood reaches the lab, results are generated in ~90 minutes, thus clinical decisions requiring BRAF status may be made within hours.

As was the case with this patient, the clinical decision on the ability to use the preferred early therapeutic, ie, BRAF/MEK inhibition, was dependent on rapid acquisition of the patient’s *BRAF* mutation status. Delay in identifying the presence of the relevant targetable variant would compel the clinician to choose the targetable therapy based on clinical judgement alone, as initiating conventional immunotherapy is in many cases financially impossible for admitted patients. Recent evidence from the DREAMseq trial, a phase III trial to compare the efficacy and toxicity of the sequence of ipilimumab/nivolumab (Ipi/Nivo) followed by dabrafenib/trametinib (Dab/Tram) to the converse sequence in treatment-naive BRAFV600-mutant patients with ECOG performance status 0 or 1 suggests that the treatment sequence beginning with the combination of Ipi/Nivo results in superior OS (11). The treatment decision made in this case predates the outcomes of the above trial to advocate beginning of treatment sequence with Ipi/Nivo followed by Dab/Tram. It is not clear from the DREAMseq data, which did not accrue patients of poor performance status, if the same survival benefit would be seen in a critically ill hospitalized patient with a immunotherapy first approach. In many centers there are administrative and financial obstacles to starting Ipi/Nivo in an inpatient setting. Given the clinical deterioration of the patient, early initiation of therapy was prudent. ctDNA-based detection of *BRAF* mutant status supported the decision to initiate targeted therapy with dabrafenib and trametinib. Of note, the patient also underwent an inpatient biopsy procedure on the same day as the peripheral blood sample for ctDNA testing was drawn. After 16 days, *BRAF* variant identification was indeterminate due to insufficient tumor in the liver biopsy. Ultimately, the ctDNA result was the only modality available to support the treatment decision, which allowed patient discharge and marked clinical improvement over the next 100 days.

Further, ctDNA-based *BRAF* variant detection has been reported to be a prognostic marker in patients with brain metastasis (12). Persistent, detectable *BRAF* variant in the patient’s plasma supported subsequent further imaging that revealed brain metastases three months after admission. The detection of new brain metastases precipitated initiation of immunotherapy with ipilimumab and nivolumab.

In conclusion, a rapid real-time PCR-based evaluation of peripheral blood could serve as a non-invasive, rapid tool to aid prompt treatment decision making in advanced melanoma in emergent situations where BRAF mutation detection utilizing NGS or IHC of the biopsy tissue is not feasible or could potentially delay clinical decision making.

## Data Availability Statement

The original contributions presented in the study are included in the article/supplementary material. Further inquiries can be directed to the corresponding author.

## Ethics Statement

The studies involving human participants were reviewed and approved by Cleveland Clinic ethics committee. Written informed consent was obtained from the next of kin for the publication of any potentially identifiable images or data included in this article.

## Author Contributions

TB, JS, JK, DE, JA, BG, DF and PF contributed to conception and writing, DF and PF contributed to finalize the manuscript. All authors approved the submitted version.

## Funding

This work and TB were partially supported by Gross Family Melanoma Registry.

## Conflict of Interest

The authors declare that the research was conducted in the absence of any commercial or financial relationships that could be construed as a potential conflict of interest.

## Publisher’s Note

All claims expressed in this article are solely those of the authors and do not necessarily represent those of their affiliated organizations, or those of the publisher, the editors and the reviewers. Any product that may be evaluated in this article, or claim that may be made by its manufacturer, is not guaranteed or endorsed by the publisher.

## Abbreviations

ICI, immune checkpoint inhibitor; PD-1, programmed cell death-1; PET-CT, positron emission tomography-CT.

## References

[1] Pessoa LS Heringer M Ferrer VP. ctDNA as a Cancer Biomarker: A Broad Overview. Crit Rev Oncol Hemat (2020) 155:103109. doi: 10.1016/j.critrevonc.2020.103109 33049662

[2] Qian C Dai N Xu M Luo H Feng Y Zhang M. ctDNA Facilitated the Diagnosis of a Patient With Synchronous Urothelial Carcinoma and Non-Small Cell Lung Cancer: Case Report. Ann Transl Med (2020) 8:1323. doi: 10.21037/atm-20-6552 33209903 PMC7661894

[3] Openshaw MR Harvey RA Sebire NJ Kaur B Sarwar N Seckl MJ. Circulating Cell Free DNA in the Diagnosis of Trophoblastic Tumors. Ebiomedicine (2016) 4:146–52. doi: 10.1016/j.ebiom.2015.12.022 PMC4776063 26981554

[4] Dharajiya NG Grosu DS Farkas DH McCullough RM Almasri E Sun Y. Incidental Detection of Maternal Neoplasia in Noninvasive Prenatal Testing. Clin Chem (2018) 64:329–35. doi: 10.1373/clinchem.2017.277517 28982650

[5] Khan M Zheng T Zhao Z Arooj S Liao G. Efficacy of BRAF Inhibitors in Combination With Stereotactic Radiosurgery for the Treatment of Melanoma Brain Metastases: A Systematic Review and Meta-Analysis. Front Oncol (2021) 10:586029. doi: 10.3389/fonc.2020.586029 33692938 PMC7937920

[6] Marczynski GT Laus AC dos Reis MB Reis RM de L Vazquez V. Circulating Tumor DNA (ctDNA) Detection Is Associated With Shorter Progression-Free Survival in Advanced Melanoma Patients. Sci Rep UK (2020) 10:18682. doi: 10.1038/s41598-020-75792-1 PMC7596487 33122747

[7] Uguen A Troncone G. A Review on the Idylla Platform: Towards the Assessment of Actionable Genomic Alterations in One Day. J Clin Pathol (2018) 71:757. doi: 10.1136/jclinpath-2018-205189 29903742

[8] Khorana AA Tullio K Elson P Pennell NA Grobmyer SR Kalady MF. Time to Initial Cancer Treatment in the United States and Association With Survival Over Time: An Observational Study. PloS One (2019) 14:e0213209. doi: 10.1371/journal.pone.0213209 30822350 PMC6396925

[9] Long GV Wilmott JS Capper D Preusser M Zhang YE Thompson JF. Immunohistochemistry Is Highly Sensitive and Specific for the Detection of V600E BRAF Mutation in Melanoma. Am J Surg Pathol (2013) 37:61–5. doi: 10.1097/PAS.0b013e31826485c0 23026937

[10] Bisschop C Elst At Bosman LJ Platteel I Jalving M Berg Avd. Rapid BRAF Mutation Tests in Patients With Advanced Melanoma. Melanoma Res (2018) 28:96–104. doi: 10.1097/CMR.0000000000000421 29232304 PMC5844592

[11] Atkins MB Lee SJ Chmielowski B Ribas A Tarhini AA Truong T-G. DREAMseq (Doublet, Randomized Evaluation in Advanced Melanoma Sequencing): A Phase III Trial—ECOG-ACRIN Ea6134. J Clin Oncol (2021) 39:356154–4. doi: 10.1200/JCO.2021.39.36_suppl.356154

[12] Behera TR Song JM Eicher DM Gastman B Farkas DH Funchain P. Circulating Tumor DNA Mutation as a Prognostic Marker in Melanoma With Brain Metastasis. J Clin Oncol (2021) 39:e21560–0. doi: 10.1200/JCO.2021.39.15_suppl.e21560

